# Pneumococcal carriage in children and their household contacts six years after introduction of the 13-valent pneumococcal conjugate vaccine in England

**DOI:** 10.1371/journal.pone.0195799

**Published:** 2018-05-25

**Authors:** Jo Southern, Nick Andrews, Pamela Sandu, Carmen L. Sheppard, Pauline A. Waight, Norman K. Fry, Albert Jan Van Hoek, Elizabeth Miller

**Affiliations:** 1 Immunisation, Hepatitis and Blood Safety Department, National Infection Service, Public Health England, London, United Kingdom; 2 Statistics, Modelling and Economics Department, Colindale, National Infection Service, Public Health England, London, United Kingdom; 3 Respiratory and Vaccine Preventable Bacteria Reference Unit, National Infection Service, Public Health England, London, United Kingdom; 4 Department of Infectious Disease Epidemiology, London School of Hygiene & Tropical Medicine, London, United Kingdom; Instituto Butantan, BRAZIL

## Abstract

**Background:**

In April 2010, 13-valent pneumococcal conjugate vaccine (PCV13) replaced PCV7 in the infant immunisation schedule in England and Wales. Despite limited serotype replacement in invasive pneumococcal disease (IPD) during the first four post-PCV13 years, non-vaccine type (NVT) IPD increased substantially in 2014/15. We undertook a carriage study in 2015/16 to help understand the reasons for this increase.

**Methods and findings:**

Families with a child aged <5 years attending a participating general practice in Gloucestershire or Hertfordshire were invited to provide nasopharyngeal swabs from all consenting members. Swabs from 650 individuals (293 under five, 73 five to twenty and 284 >twenty years) were cultured and serotyped for *Streptococcus pneumoniae*. Results were compared with those from three previous household studies conducted in the same populations between 2001 to 2013, and with the serotypes causing IPD to estimate case-carrier ratios (CCRs). Overall carriage prevalence did not differ between the four carriage studies with reductions in vaccine-type carriage offset by increases in NVT carriage. While no individual NVT serotype showed an increase in CCR from 2012/13, the composition of the serotypes comprising the NVT group differed such that the overall CCR of the NVT group had significantly increased since 2012/13. Carriage of two PCV13 serotypes, 3 and 19A, was found in 2015/16 (3/650 = 0.5% and 2/650 = 0.3% respectively) with no overall reduction in carriage prevalence of PCV13-7 serotypes since 2012/13, though 6C prevalence, a vaccine-related serotype, had reduced from 1.8% in 2012/13 to 2/648 (0.3%) in 2015/16, p = 0.013.

**Conclusions:**

There was continuing evolution in carried NVTs six years after PCV13 introduction which, in addition to being vaccine-driven, could also reflect natural secular changes in certain NVTs. This poses challenges in predicting future trends in IPD. Elimination of carriage and disease due to serotypes 3 and 19A may not be achieved by PCV13.

## Introduction

*Streptococcus pneumoniae* frequently colonises the human nasopharynx though most carriers remain asymptomatic. However, sometimes the bacteria spread locally causing non-invasive, mucosal infections such as sinusitis and otitis media or, rarely, more serious invasive disease, including septicaemia, meningitis and bacteraemic pneumonia[1]. The risk of developing invasive pneumococcal disease (IPD) during colonisation depends both on host susceptibility and invasiveness of the pneumococcus[1]. Of the >90 known pneumococcal serotypes [1], the seven serotypes (4, 6B, 9V, 14, 18C, 19F and 23F) included in the 7-valent pneumococcal conjugate vaccine (PCV7) together accounted for 74% of IPD cases in children under 2 years of age in England and Wales prior to PCV7 introduction [2] a similar percentage to their overall prevalence in carriage [3].

PCV7 was introduced into the routine infant immunisation programme in the United Kingdom (UK) in September 2006 using a 2,4, 13 month schedule with a catch up to 2 years of age. Within four years of its introduction there was a profound effect on PCV7-type IPD in all age groups with a 98% reduction in children under 2 years and an 81% reduction in adults aged 65 years and over [4]. This reflected the ability of the vaccine not only to protect vaccinated children against IPD but also against carriage of vaccine serotypes [5, 6, 7] which generated herd immunity in unvaccinated cohorts. Reducing carriage of PCV7 serotypes led to replacement colonisation by serotypes not included in PCV7 with a consequent increase in non-PCV7 e type IPD in countries with established PCV7 programmes [8]. A post-PCV7 carriage study in England in 2008/9 showed that overall carriage rates remained largely unchanged with the reduction in carriage of PCV7 serotypes offset by an increase in other serotypes [7]. The replacing serotypes that were not covered by the 13 valent vaccine (PCV13) had generally low case-carrier ratios, comprising 72% of carriage isolates but only 34% of IPD; in contrast the additional serotypes in PCV13 had a greater invasive potential comprising only 19% of carriage isolates but 48% of IPD. This suggested that even if introduction of PCV13 resulted in complete carriage replacement with non-PCV13 serotypes there would still be an overall reduction in IPD [7].

PCV13 was replaced by PCV7 in the UK in April 2010 without a catch up. A third carriage study carried out in 2012–13 in the same population as the studies by Hussein et al [3] and Flasche et al. [7] showed similar overall carriage rates with non-PCV13 serotypes comprising 96% of carried isolates but only 59% of IPD cases[9]. This supported the expectation that despite full serotype replacement in carriage the overall lower invasiveness potential of the non-PCV13 serotypes would limit the extent of replacement IPD. Trends in IPD during the first four years of the PCV13 programme in England and Wales were consistent with this expectation showing a progressive decline in overall IPD despite some replacement disease with non-vaccine serotypes (NVTs) [10]. However, in the epidemiological year July 2014 to June 2015 a sharp increase in NVT IPD was observed that involved a range of serotypes, particularly in individuals aged 15 years and over[11]. This increase in NVT IPD has continued into the 2016/2017 epidemiological year and in those aged 65 years and over has offset the reduction in vaccine-type IPD since PCV13 introduction such that the incidence of IPD overall returned to the same level as in 2008/10 before the change to PCV13. Serotypes 3 and 19A have continued to cause IPD and in 2016/17 were respectively the third and sixth most common [11].

We therefore undertook a fourth carriage study in 2015/16 to document the serotypes colonising the nasopharynx and, by comparing with IPD data for the same period, to examine their invasiveness potential and whether this had changed over the 15 year period in which the four studies were conducted.

## Methods

### Study population

The study was carried out in the same population and using the same methodology as the three earlier studies by our group [3,7,9] Briefly, families with at least one child aged 1–5 years and registered at a participating general practice (GP) in Hertfordshire or Gloucestershire were invited to take part. Written informed consent was obtained; those with any of the following were excluded: moderate to severe cerebral palsy or other debilitating condition; syndromes and neurological disorders affecting swallowing; ear, nose and throat disorders affecting local anatomy for swabbing; confirmed or suspected immunodeficiency (congenital or acquired) or receiving immunosuppressive therapy.

## Laboratory methods

Nasopharyngeal swabs were taken by trained nurses and placed directly into a broth medium containing skim milk, tryptone, glucose, and glycerin (STGG). Those taken in Hertfordshire were couriered the same day in cool storage to the Respiratory & Vaccine Preventable Bacteria Reference Unit (RVPBRU) Microbiology Services at Public Health England, Colindale, stored overnight at 2–8°C and frozen the next morning at −80°C. Samples collected in Gloucestershire were stored locally at the Gloucester Vaccine Evaluation Unit at −80°C and transferred to RVPBRU at the end of the study on dry ice. On receipt, samples were stored at −80°C.

The samples were cultured for *S*. *pneumoniae* at the end of the study by thawing of the STGG broth, vortexing and removal of 50ul sample. The 50ul inoculum was used as the primary pool on each plate for streaking using a loop. For each STGG sample replicate plates of Columbia blood agar and Streptococcus-selective Blood agar (COBA) were inoculated.^.^ Any colonies resembling pneumococcus were subcultured for DNA extraction and whole genome sequencing (WGS) and organism identification and serotype determination performed by bioinformatics methods as detailed in Kapatai et al 2016[12] with the exception of serotypes within serogroup 24 which were identified by conventional methods using antisera as previously described [3, 7, 9]. If colonies of differing morphologies were observed on the plates these were picked and subcultured separately before DNA extraction and sequencing. Carriage density was assessed by reference to the streaking pattern where + indicated growth only present in the inoculation pool, 2+ had growth present in second streak etc. to a maximum of 4+. Where the pool growth was scanty an assessment of the number of individual colonies was made.

## Statistical analyses

Descriptive analyses of the features of participating households (HH) and the age breakdown and gender of participants were conducted (Table 1). For each age group and overall, the percentage of individuals carrying a pneumococcus and exact 95% confidence intervals (CIs) were calculated based on the following serotype groupings: NVT, PCV7, PCV13, and the additional 6 serotypes in PCV13 compared with PCV7 (PCV13-7). Non-typeable pneumococcal isolates were categorized as NVT. Data analysis was in Stata Version 13 (StataCorp, Texas).

**Table 1:** Overview of numbers of participants recruited, their demographic features and household (HH) structures in the 2001/02, 2008/09, 2012/2013 and current carriage studies.

	2001/02	2008/09	2012/13	2015/16
**Number of Participants**	488	382	683	657
**Number of Swabs taken**	3868	382	683	650
**Number of Participants <5 years (%)**	180 (37)	192 (50)	277 (41)	293 (45)
**Number of Participants 5–20 years (%)**	71 (15)	57 (15)	112 (16)	73 (11)
**Number of Participants 20+ years (%)**	237 (49)	133 (35)	294 (43)	284 (44)
**Number of Proportion female**	53.0%	56.4%	57.1%	62%
**Number of HH**	121	146	217	244
**Median HH size (range)**	4 (2–7)	4 (3–7)	4 (2–8)	3 (2–7)
**Median # adults in HH (range)**	2 (1–5)	2 (1–5)	2 (1–4)	2 (1–3)
**Median # children in HH (range)**	1 (1–3)	2 (1–4)	2 (1–5)	2 (1–5)
**Proportion of smoke free HH**	66.9%	81.0%	84.3%	79.0%
**Recent antibiotic use (% of swabs)**	338 (8.7)	4 (1.0)	Analysis of 12/13 data excluded children with recent antibiotic use	6 (0.9)

For estimation of the combined direct and indirect effects of vaccination on carriage over time, carriage rates and 95% CIs by age were compared with those previously described by Van-Hoek et al [9] for the three earlier surveys using Fisher’s exact test. A model was generated to obtain adjusted odds ratios of carriage of the three cross sectional surveys compared to the longitudinal survey of 2001/02 using a generalised estimating equation (GEE) model with exchangeable correlation structure and factors for study period, age in years, gender, smoking status in the household, number of children and adults in the household, and antibiotic usage. For this analysis age was stratified by <5, ≥5 years and overall.

The proportion of individuals carrying a pneumococcus was compared by quarter (December-February, March to May, June to August and September–November). For individuals aged < 60 years, carriage density by quarter was compared with overall cases of IPD for the same periods.

Serotype-specific case-carrier ratios (CCRs) per 100,000 population were calculated using serotype-specific carriage prevalence × the population of England and Wales as the denominator with 95% confidence intervals based on the CIs for the carriage prevalence. For the numerator, IPD case numbers were obtained from laboratory reports to the national IPD surveillance data base for the corresponding study period and age- group, adjusted for the proportion not serotyped as previously described [9]. CCRs were compared with those derived for the 2012/13 carriage study [9]. As before, those aged ≥60 years were excluded due to low carriage prevalence.

Simpson’s index for diversity was calculated to assess the change in diversity in the pneumococcal population associated with vaccination [13] with results compared to the 2012/13 post-PCV13 carriage data.

## Governance

The study was approved by the NHS Health Research Authority and the London—Fulham Research Ethics Committee (reference 15/LO/0458), and was registered on clinicaltrials.gov (reference NCT02522546).

## Results

### Study population

Between July 2015 and June 2016 a total of 657 participants were recruited from 244 households, of whom 650 provided a swab; the number of individuals swabbed in each of the study sites was similar (323 in Hertfordshire and 327 in Gloucestershire) and there was no difference in median ages (18.4 years in Hertfordshire,19.5 years in Gloucestershire, p = 0.80, Kruskal-Wallis test).

The age distribution of participants in the 2015/16 survey was broadly similar to that in the three earlier surveys (Table 1). Of the 362 children in the 2015/16 survey aged less than 12 years and therefore eligible to receive either PCV7 or PCV13 for primary or catch-up immunisation, only 13 (3.6%) had not received at least one PCV7 (n = 7) or PCV13 (n = 6) dose; the majority (295) had received 3 doses of PCV13, 40 had had 3 doses of PCV7 or a mixed schedule and 14 less than 3 doses of any PCV. Household characteristics were similar between the three studies though with a median number of members of three in 2015/16 compared with four in the earlier studies (Table 1).

## Carriage of vaccine and non-vaccine serotypes

The prevalence of carriage of any pneumococcus, overall or within each age group, did not differ significantly between the four studies (Table 2). In the 2015/16 study overall carriage prevalence was similar at the Hertfordshire and Gloucestershire sites (91/323, 28.2% and 89/327, 27.2% respectively) as was the serotype distribution, p = 0.74 exact test (see S1 Fig).

**Table 2:** Number of positive NVT, PCV7, the extra 6 serotypes in PCV13 and All (including non-typeable) carriage isolates in 2012/13 and the overall carriage rate for 2001/02, 2008/09, 2012/13 and 2015/16. The proportion for 2001/02 was calculated accounting for multiple testing of the participants.

		NVT (95% CI)^1^	PCV7 (95% CI)	Extra 6 PCV13 serotypes (95% CI)	ALL (95% CI)
**<5 years**	**Participants 15/16 (n = 293)**	150	0	3	152
	**Proportion 15/16**	51.1% (45.3–57.0)	0.0% (0.0–1.3)	1.0% (0.2–3.0)	51.9% (46.0–57.7)
	**Proportion 12/13**	46.9% (41.1–53.5)	0.4% (0.0–2.0)	0.4% (0.0–2.0)	47.7% (41.8–53.5)
	**Proportion 08/09**	37.0% (30.5–44.0)	4.2% (2.1–8.0)	9.9%(6.4–14.9)	51.0% (44.0–58.0)
	**Proportion 01/02**	8.5% (6.4–11.1)	31.9% (28.1–36.1)	8.0% (6.0–10.6)	48.4% (44.1–52.7)
**5–20 years**	**Participants 15/16 (n = 73)**	18	0	2	20
	**Proportion 15/16**	24.7% (15.3–36.1)	0.0% (0.0–4.9)	2.7% (0.3–9.5)	27.4% (17.6–39.1)
	**Proportion 12/13**	19.6% (13.3–28.0)	0.9% (0.0–4.9)	1.8% (0.5–6.3)	22.3% (15.6–30.9)
	**Proportion 08/09**	22.8% (13.8–35.2)	0.0% (0.0–6.3)	5.3% (1.8–14.4)	28.1% (18.1–40.8)
	**Proportion 01/02**	8.5% (5.6–12.8)	10.0% (7.5–13.4)	2.0% (1.0–4.1)	21.1% (16.5–26.5)
**>20 years**	**Participants 15/16 (n = 284)**	8	0	0	8
	**Proportion 15/16**	2.8% (1.2–5.4)	0.0% (0.0–1.3)	0.0% (0.0–1.3)	2.8% (1.2–5.5)
	**Proportion 12/13**	3.1% (1.6–5.7)	0.0% (0.0–1.3)	0.3% (0.0–1.9)	3.4% (1.9–6.1)
	**Proportion 08/09**	6.0% (3.1–11.4)	2.3% (0.8–6.4)	1.5% (0.4–5.3)	9.8% (5.8–16)
	**Proportion 01/02**	1.9% (1.3–2.9)	4.0% (3.0–5.4)	1.6% (1.0–2.5)	7.6% (6.1–9.4)
**All**	**Participants 15/16 (n = 650)**	176	0	5	180
	**Proportion 15/16**	27.0% (23.7–30.7)	0.0% (0.0–0.6)	0.7% (0.3–1.8)	27.7% (24.3–31.3)
	**Proportion 12/13**	23.6% (20.5–26.9)	0.3% (0.1–1.1)	0.6% (0.2–1.5)	24.5% (21.4–27.8)
	**Proportion 08/09**	24.1% (20.1–28.6)	2.9% (1.6–5.1)	6.3% (4.3–9.2)	33.2% (28.7–38.1)
	**Proportion 01/02**	5.2% (4.2–6.5)	15.2% (13.2–17.4)	4.1% (3.2–5.2)	24.4% (21.9–27.1)

^1^ 6C and non-typeable pneumococci are included in the non-vaccine types. Multiple carriage was detected in 5 individuals. These had 10A/21, 22F/Non-typeable, 23B/Non-typeable, 3/23B and 9N/6C. When assessing the proportion of individuals who carried any VT or NVT serotype, multiple carriage episodes in individuals who carried both a VT and NVT serotype are included. For this reason adding up the number of VT and NVT carriers is one more than the total for carrying any serotype. Non-typeable isolates were counted as NVTs for this analysis to be consistent with the 2012/13 analysis.

No carriage of PCV7 serotypes was detected in 2015/16, continuing the progressive reduction seen since the pre-PCV era. For the extra six serotypes included in PCV13, overall carriage prevalence in 2015/16 was similar to that in 2012/13, 0.7% (95% CI 0.3–1.8) and 0.6% (0.2–1.5) respectively. When compared with the 2001/02 pre-PCV baseline using the GEE model the overall odds of carriage of any pneumococcus was not significantly different from 1 in any of the post- PCV surveys (Table 3); similar results were obtained when stratified by age, with the exception of a marginally significant reduction in carriage prevalence in those aged 5 years and over in 2015/16 (odds ratio 0.55, 0.30–0.99) (Table 3). When stratified by serotype grouping results showed that compared to 2001/02 there was a reduction in PCV7 and PCV13-7 serotypes which was offset by the progressive increase in the carriage prevalence of NVTs. Compared to 2012/13 there was a small non-significant increase in prevalence of NVTs in 2015/16 as shown in Table 2; the OR for carriage of a NVT (all ages) in 2015/16 compared with 2012/13 was 1.14 (0.86–1.52), p = 0.37.

**Table 3:** Adjusted odds ratios* for the carriage of PCV7, PCV13, non-PCV and any serotypes in 2008/09, 2012/13 and 2015/16 to carriage in the longitudinal study in 2001/02 by age group using GEE.

Serotype group	<5 years (95% CI)			≥5 years (95% CI)			All (95% CI)		
	2008/9	2012/13	2015/16	2008/9	2012/13	2015/16	2008/9	2012/13	2015/16
PCV7	0.07 (0.03–0.17)	0.01 (0.00–0.09)	0.00 -	0.29 (0.04–2.41)	0.04 (0.01–0.32)	0.00 -	0.08 (0.04–1.19)	0.02 (0.00–0.08)	0.00 -
Extra 6 in PCV13	1.19 (0.63–2.27)	0.04 (0.01–0.34)	0.12 (0.03–0.45)	0.94 (0.17–5.33)	0.14 (0.03–0.56)	0.09 (0.02–0.47)	1.21 (0.71–2.05)	0.11 (0.05–0.35)	0.13 (0.06–0.36)
Non- PCV13	5.28 (3.23–8.63)	8.41 (5.31–13.3)	9.73 (6.12–15.5)	5.28 (1.72–16.2)	3.03 (1.46–6.29)	3.10 (1.42–6.75)	4.44 (2.93–6.74)	5.65 (3.94–8.10)	6.45 (4.47–9.32)
Any	0.99 (0.67–1.47)	1.05 (0.73–1.50)	1.20 (0.83–1.74)	1.47 (0.59–3.65)	0.58 (0.34–1.01)	0.55 (0.30–0.99)	0.99 (0.69–1.41)	0.84 (0.63–1.11)	0.93 (0.69–1.25)

*Adjusted for age, gender, smoking in the household, number of children and adults in the household and antibiotic use.

**Fig 1:**
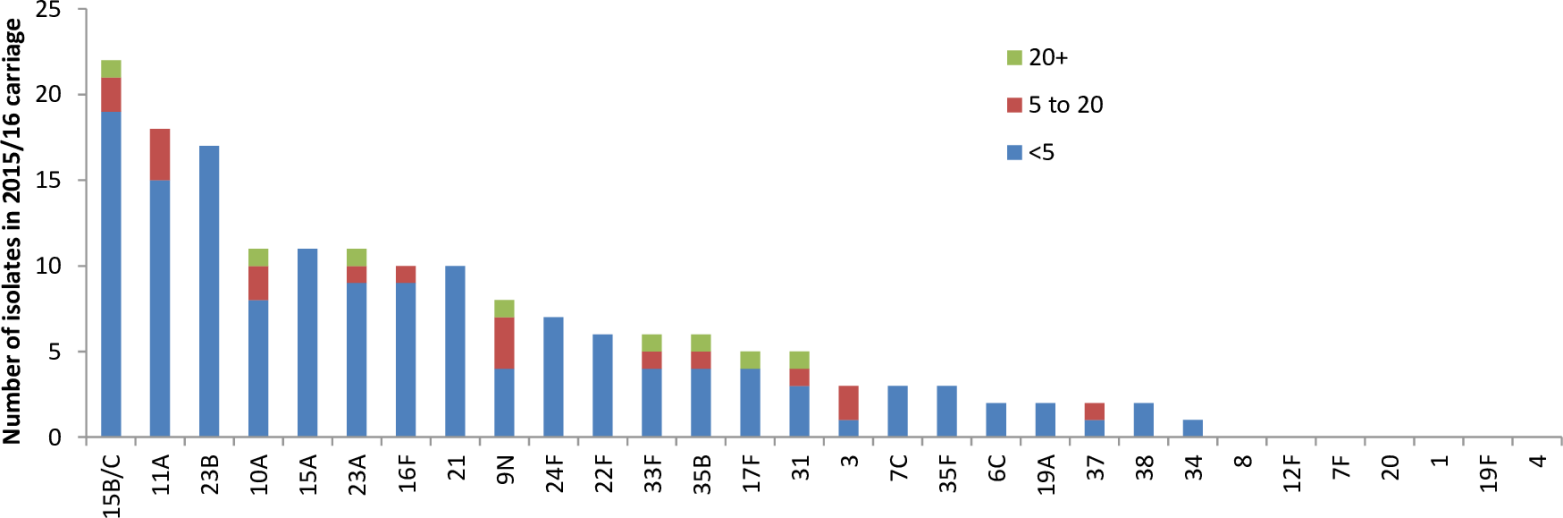
Number of serotypes isolated in the 2015/16 survey by age group. Non typeable pneumococcal isolates excluded.

**Fig 2:**
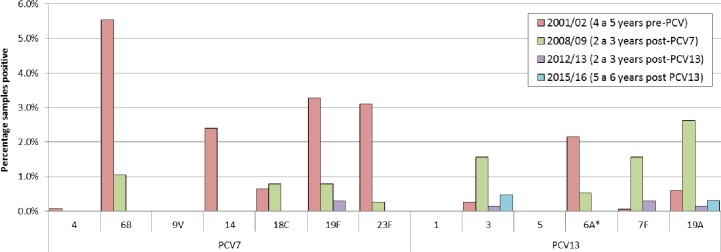
The carriage of PCV13 types in the four consecutive carriage studies performed in the two study sites. The study in 2001/02 was a longitudinal study the three subsequent studies cross-sectional. *The percentage of samples positive for serotype 6A in 2001/02 was post-hoc corrected for 6C (6A is 68% of original) as described in Flasche et al. [7].

Fig 1 shows the prevalence of the individual serotypes detected in the 2015/16 survey by age group. Only two serotypes covered by PCV13 were detected in carriage, 19A and 3, though at a low prevalence (2/650 = 0.3% and 3/650 = 0.5%, respectively) compared with the NVTs. The two 19A carriers had both received 3 doses of PCV13, as did the only ST 3 carrier who was eligible to have received the full course. Fig 2 compares the prevalence of PCV7 and PCV13-7 serotypes in each of the four carriage surveys. Serotypes 1, 5 and 9V were not detected in any of the surveys. Of the six remaining PCV7 serotypes, 19F was still detectable in the 2012/13 survey, six to seven years after PCV7 introduction but not in 2015/16. (Fig 1). Carriage of 6A, a PCV13-7 serotype had already fallen from 2.2% in the 2001/02 survey to 0.5% in 2008/09 before PCV13 introduction consistent with cross protection from the 6B component of PCV7. No 6A carriage isolates were found in the post–PCV13 surveys.

**Fig 3:**
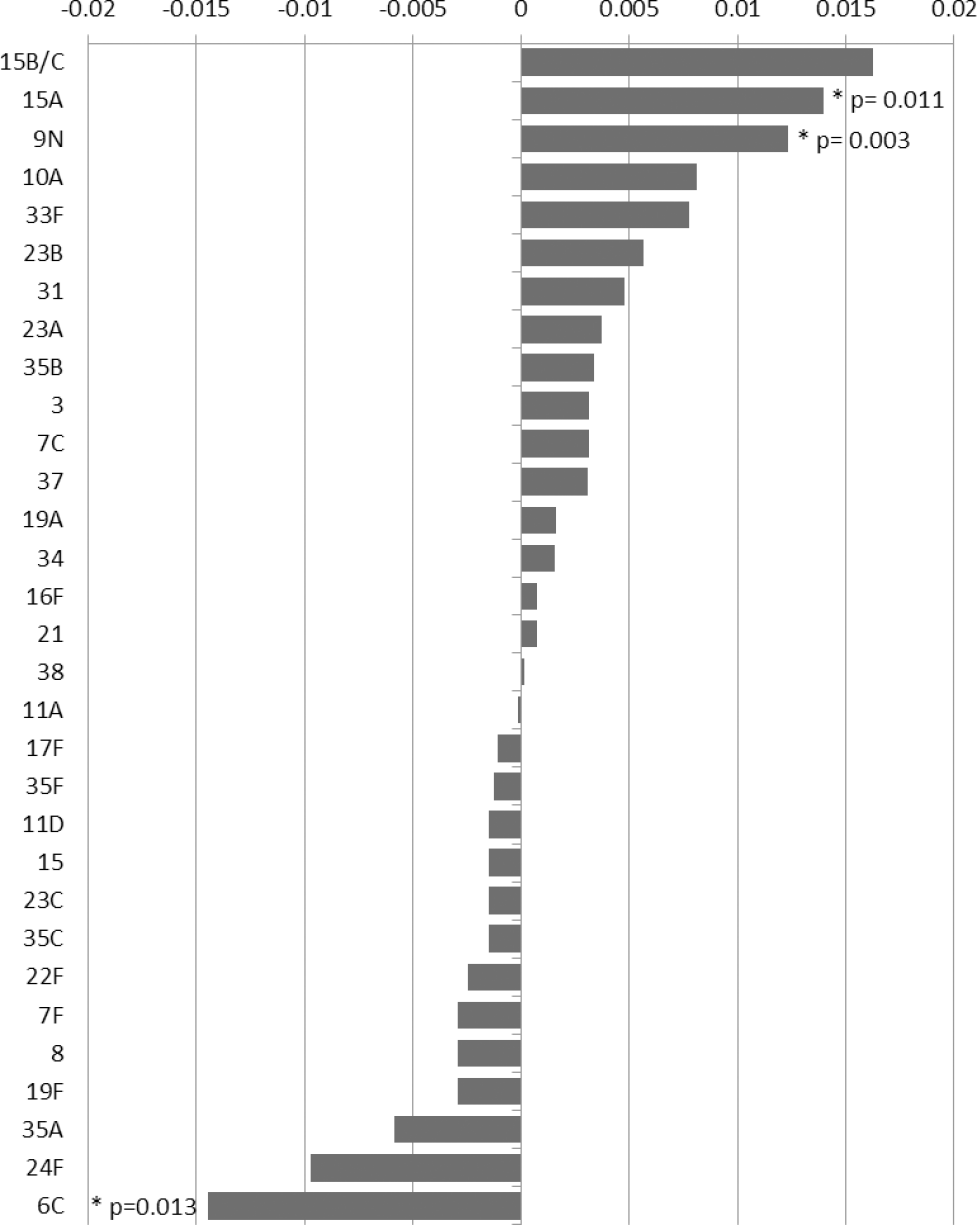
Change in prevalence of serotypes (measured as the difference in proportions of all swabs taken that were culture positive for that serotype) in those aged <60 years between the 2012/13 and 2015/16 carriage surveys. P values shown for serotypes where the changes was <0.05.

The difference between the prevalence of individual serotypes detected in the 2012/13 and/or the 2015/16 studies is compared in Fig 3. Of the serotypes showing evidence of a decline, the greatest reduction was in the vaccine-related serotype 6C which was the fifth most common NVT serotype carried in the 2012/13 survey[9]. Prevalence of 6C declined from 12/671 (1.8%) in 2012/13 to 2/648 (0.3%) in 2015/16, p = 0.013; the prevalence of 6C in 2012/13 was similar to that in 2008/09 (2.1%, p = 0.814) with a possible increase post PCV7 compared to the pre PCV7 survey when prevalence was 1.0% (p = 0.67). Serotype 24F, another common NVT carried in the 2012/13 survey also declined though not significantly. Serotypes that showed a significant increase in prevalence between the 2012/13 and 2015/16 surveys were 15A and 9N.

Simpsons diversity index for the 2015/16 survey was 0.94 (95% CI: 0.93–0.96) and the same as in the 2012/13 survey (0.94, 95% CI 0.93–0.95). Both were significantly higher than in the pre-PCV period for which the diversity index for the 2001/2002 samples was 0.91 (95% CI: 0.90–0.92).

## Comparison of serotypes in carriage and IPD

**Fig 4:**
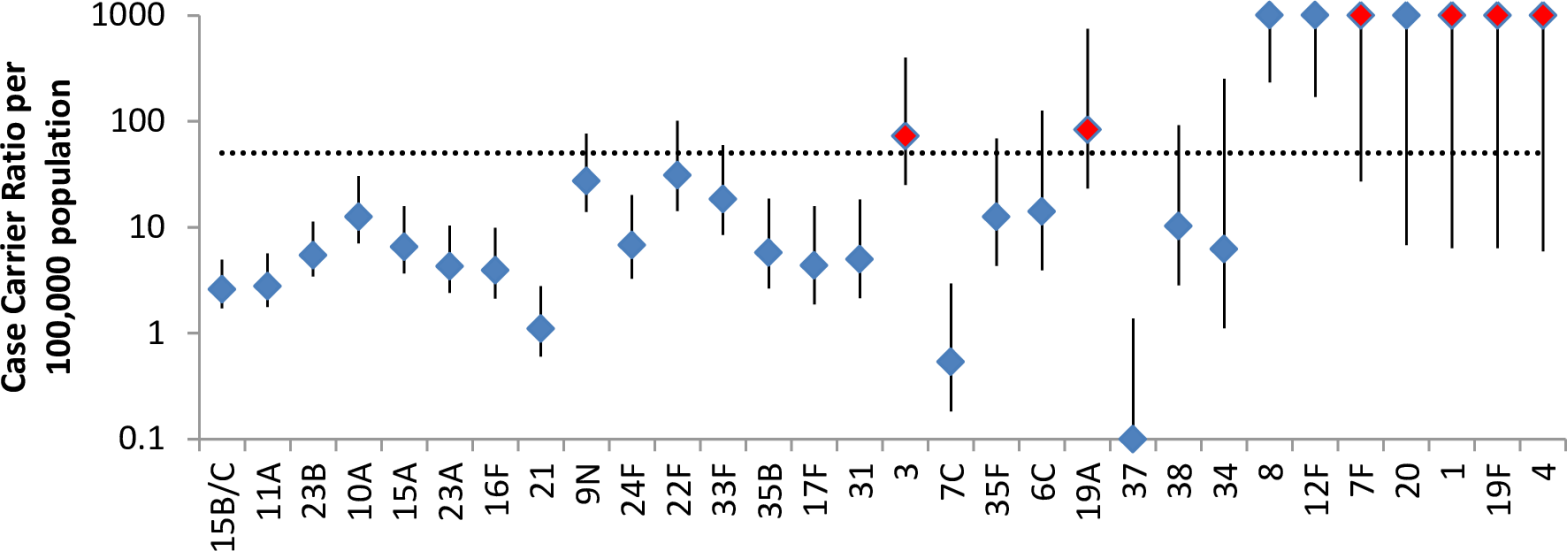
Case carrier ratios in 2015/16. The bar represents the 95% CI and vaccine serotypes are shown in red. Data includes just those aged <60 and serotypes with no carriage but at least 10 IPD isolates in 2015/16 in those aged <60 years.

The proportion of carriage pneumococci that were vaccine and non-vaccine type compared with the proportion in IPD is shown in Table 4. Despite comprising only 2.8% of carried isolates, PCV13 serotypes still accounted for 17.6% of IPD cases in the same period, a reduction from 40.9% in the 2012/13 study. Fig 4 shows the CCRs of the serotypes carried in the 2015/16 survey; for those serotypes that were not detected in carriage but comprised at least 10 IPD cases in individuals under 60 years during the same time period July 2015 to June 2016 a lower end to the 95% confidence interval for the CCR based on an observed carriage prevalence of zero has been estimated. Most of the NVTs had relatively low CCRs compared with the PCV 13 serotypes with the exception of 8, 12F and 20 none of which were detected in carriage but were found in IPD; in 2012/13 these three serotypes were also not found in carriage but were a common cause of IPD (S1 Table).

**Table 4:** The proportion of carried pneumococci and IPD cases caused by serotypes covered and not covered by PCV7, the additional three serotypes in PCV10 and the additional three in PCV13 according to study year.

	2015/16		2012/13		2008/09		2001/02	
	Carriage (%)	% in IPD	Carriage (%)	% in IPD	Carriage (%)	% in IPD	Carriage (%)	% in IPD
**PCV7**	0 (0.0)	2.4	2 (1.2)	2.9	11 (8.7)	15.2	605 (62.2)	55.9
**+PCV10**	0 (0.0)	3.7	2 (1.2)	25.2	6 (4.7)	32.6	2 (0.2)	10.2
**+PCV13**	5 (2.8)	11.5	2 (1.2)	12.8	18 (14.2)	15.8	155 (15.9)	8.9
**Rest**	176 (97.2)	82.4	160 (96.4)	59.1	92 (72.4)	36.4	210 (21.7)	25.0

**Fig 5:**
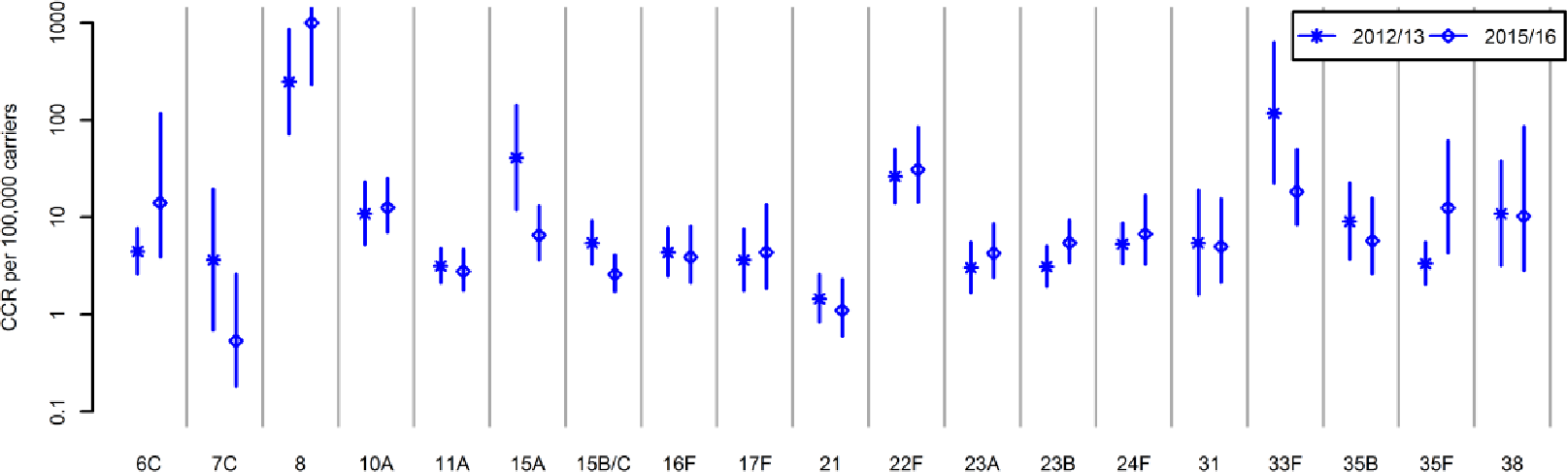
Case carrier ratios of non-vaccine serotypes in the 2012/13 survey [9] compared with the 2015/16 survey. Fig excludes non-vaccine serotypes shown in Fig 4 but with no carriage detected in the 2012/13 study (12F, 9N, 20, 34, 37 with cases of IPD respectively 98, 47, 24, 3, 2 S1 Table).

When compared with the 2012/13 study, none of the NVTs showed a substantive increase in CCR in 2015/16 (Fig 5). When comparing the CCR of all NVTs combined between the 2012/13 and 2015/16 surveys there was a significant increase, 10.9 (9.5–12.7) versus 16.8 (14.8–19.3). Despite this increase, the overall CCR of PCV13 serotypes in 2015/16 was still significantly higher (118.9, 51.2–365.4). For the two PCV13 serotypes detected in both the 2012/13 and 2015/16 surveys (3 and 19A) the CCRs were not significantly different between surveys (data not shown).

## Carriage prevalence, carriage density and IPD incidence

The proportion of subjects who were carrying a pneumococcus did not vary by quarter (p = 0.20): Dec-Feb: 21/79 (26.6%), Mar-May: 33/104 (31.7%), Jun-Aug: 35/162 (21.6%) and Sep-Nov: 91/305 (29.8%). Carriage density showed no difference by serotype (p = 0.72) but some evidence of a difference by age (p = 0.05) where the proportion positive in the 2^nd^ or subsequent quadrant was 94/152 (62%) in those aged <5 years, 9/20 (45%) in those aged 5 to 20 years and 2/8 (25%) in those aged > = 20 years. There was also evidence (p = 0.04) that density was higher in the winter months (Dec-Feb) when 21/26 (81%) were positive in the 2^nd^ or subsequent quadrant compared to 19/33 (58%), 16/35 (46%) and 52/91 (57%) in the other quarters (Mar-May, Jun-Aug, Sep-Nov). While the highest number of IPD cases was also reported in the same Dec-Feb quarter (781), the correspondence between carriage density and IPD cases was less clear in the other three quarters (Mar-May 744, Jun-Aug 358 and Sep-Nov 438).

## Discussion

Trends in the incidence of IPD reflect changes in the prevalence and inherent invasiveness of the pneumococci carried in the nasopharynx. Our 2015/16 carriage study was conducted to try to understand the carriage dynamics associated with the sharp increase in NVT IPD that was observed four years after the change from PCV7 to PCV13 in England and Wales. We found that while there was no major change in overall carriage prevalence of NVTs nor in the invasiveness potential of any individual NVT, the case carrier ratio of the NVTs as a group had significantly increased since 2012/13. This resulted from a change in the serotype distribution of the NVTs carried in the nasopharynx which appeared to have resulted in a serotype mix with an overall higher case carrier ratio.

The continued evolving competition between the serotypes carried in the nasopharynx some five to six years after PCV13 introduction was evidenced by the high value of Simpsons diversity index in 2015/16, which was the same as in 2012/13 [9]; both were significantly higher than the pre-PCV7 value, as was the value in the 2008/9 survey carried out three to four years after PCV7 introduction [7]. While these continuing NVT changes may reflect vaccine-driven evolution, there was no further reduction in overall carriage prevalence of PCV13 serotypes since the 2012/13 study, the disappearance of serotypes 19F and 7F being offset by small increases in the prevalence of 19A and 3. Serotype 6C, a prevalent serotype in the 2012/13 carriage study [9] and with a relatively low case carrier ratio, has declined significantly since 2012/13, likely reflecting the cross protection from the 6A component of PCV13 as demonstrated in immunogenicity studies [14]. The change in prevalence from 1.8% in 2012/13 to 0.3% in 2015/16, could not be wholly responsible for the overall increase in CCR of NVTs in 2015/16 but may have contributed to continued vaccine-driven evolution. Co-circulation of influenza could potentially increase the invasiveness of carried pneumococci but the 2015/16 influenza season in England was similar to that in 2012/13 when the earlier carriage study was conducted, and also similar to the influenza seasons in the intervening years [15].

Natural secular changes in the incidence of IPD due to certain serotypes do occur as illustrated by the significant decline in the incidence of IPD due to serotype 1 that increased before the introduction of PCV7 in England and Wales and fell sharply thereafter, despite evidence of vaccine-driven replacement occurring with other serotypes [4]. Large secular fluctuations in the incidence of serotype 1 IPD have also been reported from Spain where there was an increase pre-PCV7 which continued thereafter [16], and in Sweden where serotype 1 incidence fell before PCV7 introduction [17]. While mainly documented in the pre-PCV era for serotypes covered by PCV13, some NVTs also exhibit long term secular trends in IPD unrelated to vaccination. For example, in Spain [16] and the Oxfordshire region[18] serotype 8 increased in the pre-PCV7 era, while serotypes 8 and 9N both decreased significantly in England and Wales in the post-PCV7 period [4].The factors driving such secular changes in IPD are currently poorly understood as little data are available on any associated changes in carriage prevalence or population susceptibility. One pre-PCV7 carriage study in Portuguese children did document periodic cycles in the carriage prevalence of some PCV7 serotypes and a few non-PCV7 serotypes with the periodicity perturbed by the introduction of PCV7 but associated changes in IPD or population susceptibility were not reported [19]. Some secular increases may be driven by increases in antimicrobial resistance, as for example the rise in 15A post-PCV7 which has continued post-PCV13 [11] and is associated with the emergence of a multidrug resistant clone [20]. Where antimicrobial resistance is not implicated, emergence of clones with enhanced invasiveness has been postulated. However, evidence of this is lacking; the pre-PCV7 increase in serotype 8 in Spain and the Oxfordshire study was not associated with any clonal changes [17, 19]^,^ and the recent increase in cases of 19A IPD in England and Wales was not associated with any phenotypic or genotypic changes [21].

Two PCV3 serotypes, 3 and 19A, were still detected in carriage in fully vaccinated children in the 2015/16 survey with no evidence of a decline since 2012/13. Persistence of serotype 3 in carriage is perhaps unsurprising given the lack of demonstrable direct protection against IPD in England and Wales with the 2+1 schedule and the rising incidence of serotype 3 since 2013/14[11, 22]. Protection against carriage, which likely requires higher antibody levels than protection against disease, is therefore not expected. Indeed the difference in prevalence of serotype 3 between the pre-PCV13 2008/9 carriage study and the current study (6/382 versus 3/650) is not significant (p = 0.08). For 19A, indirect protection against IPD has been shown [4] and the reduction in carriage prevalence of 19A from 10/382 in 2008/09 to 2/650 in 2015/16 is significant (p = 0.001, ad hoc analysis). However the direct protection against IPD in England and Wales was relatively low compared with other PCV13 serotypes (67%, 95% CI 33–84) [23] so protection against carriage is also likely to be lower than for other serotypes. Persistence in carriage of serotype 3 and 19A and also 19F has been demonstrated in a study in Massachusetts three to four years post-PCV13 introduction [23] and a carriage prevalence of 12.5% for 19F, 4.5% for 6A and 1.1% for 19A was found in a study in a children’s hospital in Missouri conducted three to six years post-PCV13 [24]. In the US a 3+1 schedule is used so persistence in carriage of serotypes 3 and 19A in the UK cannot be attributed to the use of the 2+1 schedule. Therefore, while transmission models predict the progressive reduction and eventual elimination of carriage of PCV13 serotypes [25, 26], this is unlikely to apply to serotype 3 and may not to apply 19A, 19F and some other serotypes for which low level transmission in the population may persist for many years post-PCV13 introduction.

Our 2015/16 survey confirmed the findings from our pre-PCV7 survey [3] that the well documented seasonal changes in IPD incidence in England and Wales are not reflected in changes in carriage prevalence. Carriage density, which did show evidence of significant seasonal changes, may be a potentially more relevant factor in determining seasonal variation in IPD incidence, though we found incomplete correspondence between density and IPD incidence Our method of assessing carriage density is somewhat subjective, although the same amount of STGG broth was added to each plate and the subculturing and streaking for all of the samples in the study was done by the same operator. Another study using the same method for assessing carriage density in children from the Navajo and White Mountain Apache populations found seasonal variations in carriage prevalence and density that correlated with increases in invasive non-pneumonia infections but not bacteraemic pneumococcal pneumonia [27]. More precise measures of carriage density, such as gene copies/mL, and separation of IPD cases by clinical presentation, may help to elucidate better the relationship between carriage density and IPD.

Our study has some limitations. First despite swabbing over 650 participants, the power to detect carriage of serotypes with a low prevalence was limited as evidenced by the failure to detect any carriage episodes for eight serotypes causing at least 10 cases of IPD in individuals under 60 years of age. Similarly our ability to detect significant changes in the carriage prevalence of individual serotypes between studies was limited. Our three previous carriage studies used conventional serotyping methods employing antisera that recognise specific capsular polysaccharides whereas the current study used WGS for serogroup/serotype identification. However, when comparing the WGS method with conventional serotyping methods the former was able to accurately predict serogroup/type for 99.1% of the typeable isolates tested ^12^. It is unlikely therefore that the difference between studies in serotyping methods has materially affected the results. The denominator for our CCR calculations used carriage data from two areas of England, Hertfordshire and Gloucestershire, whereas the IPD data was for all England. It is possible that carriage prevalence in these two areas is not nationally representative, However carriage prevalence in the two sites though geographically and economically different [28] was very similar as was the serotype distribution. When calculating CCRs, ideally the serotype-specific incidence of new carriage episodes should form the denominator, since invasion, if it happens, is thought to occur shortly after carriage acquisition. In a cross sectional prevalence study carriage duration will also affect the results, although this only matters when comparing serotypes with substantially different durations of carriage [29]. The strength of our four sequential studies is that they have taken place in healthy children and their household contacts from the same geographical locations in England over a 15 year period using the same recruitment procedures and analytic techniques, thus strengthening the comparisons made between the different studies.

In conclusion, our study shows evidence of continued evolution of the NVTs serotypes inhabiting the nasopharynx some six years after PCV13 introduction, with the recent changes in serotype distribution resulting in an overall increase in invasiveness of the NVT group since 2012/13. This continued evolution of NVTs may be driven in part by other factors such as natural secular changes in carriage prevalence, possibly with associated population susceptibility changes, and poses problems for transmission models that assume all post-PCV changes are vaccine-driven and use pre-PCV data to estimate the CCR of NVTs as a group [27]. Our study also demonstrated continued circulation of two of the additional serotypes in PCV13 (3 and 19A) against which PCV13 is least effective. This also has implications for transmission models that group PCV13-7 serotypes together and predict eventual vaccine-driven elimination. In such models it may be more appropriate to consider serotype 3 as part of the NVT group.

## Supporting information

S1 Table Pneumococcal surveillance data from 2012–13. (XLSX)

S1 Fig Serotype distribution by study site. No significant differences between sites in serotype distribution p = 0.74 exact test).
(DOCX)
