# Supplementary material for: Pneumococcal carriage in children and their household contacts six years after introduction of the 13-valent pneumococcal conjugate vaccine in England
Source: PLoS One. 2018 May 25;13(5):e0195799. doi: 10.1371/journal.pone.0195799 (PMC5969732; doi:10.1371/journal.pone.0195799)

Figure S1. Serotype distribution by study site. No significant differences between sites p=0.74 exact test).


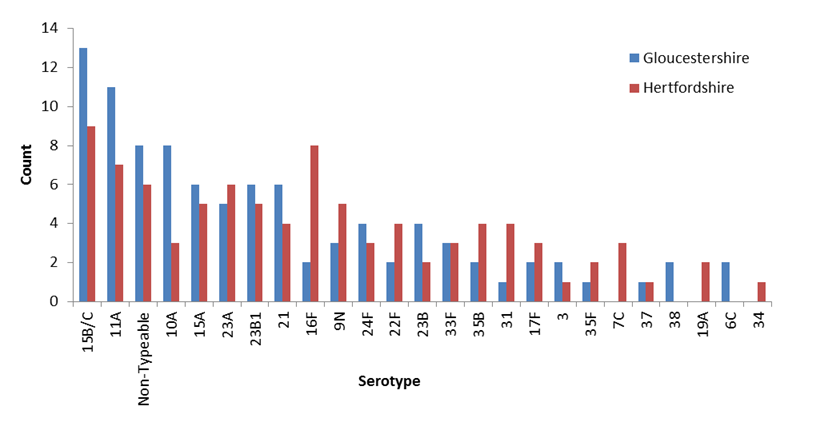

Supplement: S1 Fig — No significant differences between sites in serotype distribution p = 0.74 exact test). (DOCX) [file pone.0195799.s002.docx]
